# Effectiveness of price limits: Evidence from China’s ChiNext market

**DOI:** 10.1371/journal.pone.0287548

**Published:** 2023-06-23

**Authors:** Bao Qi

**Affiliations:** School of Finance, Shanghai University of Finance and Economics, Shanghai, China; Universiti Malaysia Sabah, MALAYSIA

## Abstract

Starting from August 24, 2020, the daily stock price limits in China’s ChiNext market have been adjusted from 10% to 20%. We use this reform to study the effectiveness of price limits in China’s stock market. We test four hypotheses about price limits: delayed price discovery, volatility spillover, trading interference, and magnet effect. Using the event study method, we examine the differences in the behavior of stock price, trading volume, and volatility before and after the reform. We confirm the delayed price discovery, volatility spillover and trading interference hypothesis of price limits, and find that these negative effects of price limits are more serious when lower limits are hit. In addition, we examine the distribution of large price movements before and after the reform and find no evidence of the magnet effect of price limits. The present research has important implications for policymakers and investors in China’s stock market.

## 1. Introduction

For a long time, China’s stock market has had daily price limits on individual stock prices. A daily price limit on a stock market refers to the maximum allowable price movement for a particular stock within a specified period. It is a mechanism designed to control extreme price fluctuations and maintain stability in the market. However, the effectiveness of price limits has been a topic of debate in the academic community. Supporters of price limits believe that it can temporarily constrain stock price volatility within a certain range, and provide investors with valuable time to calm down and digest market information. It helps alleviate investors’ overreaction, and improve stock pricing efficiency [1–4]. For these reasons, apart from the stock market in China, stock markets in South Korea, Japan, Thailand, Taiwan, and some other countries have also imposed different degrees of restrictions on daily stock price fluctuations [2, 5–7]. However, opponents of price limits argue that it is generally ineffective. The imbalance between supply and demand drives stock price fluctuations. If the equilibrium price exceeds the limit price, the stock trading and price discovery process will be forced to be delayed until the following trading day, which increases the volatility of stock prices on the following trading day and affects the liquidity of the stock [8–13].

The reason why the effectiveness of price limits is controversial is that it is impossible to find two markets that are exactly the same except for the price limits, so it is difficult to isolate the impact of price limits from many other interfering factors. We utilize a reform that occurred in China’s ChiNext market in 2020 to reevaluate the effectiveness of price limits. China’s ChiNext market is a NASDAQ-style securities trading market, established in 2009. Since its inception, the ChiNext market has implemented a 10% daily stock price limit, which means that stock prices can only fluctuate within a range of 10% from the previous day’s closing price. On August 24, 2020, the daily price limit of stocks in China’s ChiNext market was adjusted from 10% to 20%. This was the first adjustment made to the price limit system in more than a decade since the ChiNext market was established. It provides a rare opportunity to study the effectiveness of price limits in China’s stock market.

Through comparing a subperiod before the reform with a subperiod after the reform, we primarily test four hypotheses related to price limits: the delayed price discovery hypothesis, the volatility spillover hypothesis, the trading interference hypothesis and the magnet effect hypothesis. First, we identify all instances of limit-hit events before the reform within a certain period and all events with price changes exceeding the 10% threshold after the reform within another specific period. By comparing the changes in stock returns, trading volume, and volatility between these two types of events in the subsequent trading days, we confirm that price limits do cause price discovery delay, volatility spillover and trading interference, which are contrary to the findings of Kim, et al. (2013) [4]. We also find that these effects are more serious when lower limits are hit than when upper limits are hit. Second, we compare the proportion of large stock price changes before and after the reform, and find no evidence for the magnet effect.

The contributions of the research are mainly reflected in several aspects. Firstly, it enriches the literature on the effectiveness of price limits in China’s stock market. Kim, et al. (2013) [4] previously studied the effects of price limits in China’s stock market using data from the period without price limits (1992–1996) and the period with price limits (1996–2000) and concluded that the price limits was effective in China’s stock market. However, their sample period covers a large time span, during which many factors that affect the stock market, such as the economic fundamentals and investor behaviour, have changed significantly. Additionally, the Chinese stock market had just started operating in 1992, and the market was highly volatile due to the inadequate regulations and rules, making it difficult to attribute the differences solely to the impact of price limits [13]. In contrast, we utilize the price limit reform that happened in China’s ChiNext market in 2020 to test the effectiveness of price limits, and set the sample selection period as six months before and after the reform. The shorter time span can ensure that other factors, such as the market’s investor structure and economic fundamentals, do not change significantly, making the conclusions of this research relatively more reliable. Unlike previous studies, this research also finds that the influence of price limits in the process of stock price rise and fall is asymmetric. Secondly, the research results have certain policy implications. In contrast to existing literature on this reform [14–17], we take a unique approach by comparing pre-reform price limit events with post-reform events that, although not reaching the actual price limit, met the pre-reform criteria based on price fluctuations. This comparative study enables us to analyze the effects of this reform from a micro-level mechanism perspective. This research provides evidence supporting the relaxation of the daily price limit range and provides a research reference for further reforms of price limits in China’s stock market.

The rest of the paper is organized as follows. The second part is an introduction to the institutional background. The third part is the literature review. The fourth part presents the research hypotheses. The fifth part presents the empirical analysis, and the final part presents the research conclusions.

## 2. Institutional background

China’s ChiNext market was established on October 30, 2009 as a securities trading market specifically designed to provide financing opportunities and growth space for start-up enterprises that are temporarily unable to be listed on the main board market. Companies listed on the ChiNext market have high growth potential, but are often newly established with small scales and less impressive performance.

As of February 2023, there are 1,241 companies listed on the board with a total market capitalization of approximately RMB 12.2 trillion. Since its inception, the ChiNext market has implemented a 10% daily stock price limit, which means that stock prices can only fluctuate within a range of 10% from the previous day’s closing price. Any orders outside this range will be deemed invalid.

In 2020, the ChiNext market underwent a reform in its stock issuance system, shifting from an approval-based IPO system to a registration-based IPO System. The pilot registration system is important for deepening the capital market reform, improving the fundamental rules of the capital market, and enhancing the function of the capital market in China. With the implementation of the registration-based IPO system, as one of the supporting reforms, the price limit system in the ChiNext market underwent a significant adjustment. On June 12, 2020, the Shenzhen Stock Exchange issued the “Special Rules on Trading on the ChiNext Market of Shenzhen Stock Exchange”, announcing that the daily price limit for the ChiNext market would be adjusted from 10% to 20%. This rule would come into effect from the first trading day of the first stock listed under the registration-based IPO system. On August 24, 2020, as the first batch of companies listed under the registration-based IPO system started trading, the price limit in the ChiNext market was officially adjusted from 10% to 20%. This was the first adjustment made to the price limit system in more than a decade since the ChiNext market was established. The ChiNext market has been known for its high volatility and limited liquidity. By widening the price limit, it is expected to reduce the frequency of trading suspension and increase market participation, ultimately benefiting the growth of the market and supporting the development of China’s capital market. The relaxation of the daily price fluctuation limit on the ChiNext market provides a rare opportunity to study the effectiveness of the price limit system in China’s stock market, which is the focus of this research.

## 3. Literature review

Price limits are implemented in some financial markets to help regulate and stabilize trading activities, prevent excessive volatility, and protect investors from extreme price movements. However, there is still controversy regarding the effectiveness of price limits. The reason why the effectiveness of price limits is controversial is that it is impossible to find two markets that are exactly the same except for the price limits, so it is difficult to isolate the impact of price limits from many other interfering factors. For these reasons existing researches have attempted to find breakthroughs in data selection and method design.

Early researches studied the effectiveness of price limits by directly comparing the stock price volatility on limit days with the volatility after the limit days [1]. However, the reliability of these results has been questioned by many scholars [8, 18, 19]. Because even in the absence of limits, the volatility of stocks will decrease after experiencing large fluctuations. Kim and Rhee (1997) [8] cleverly used the comparison between stocks on the Tokyo Stock Exchange that had already reached the limits and stocks that had a larger daily price change but had not yet reached limits to study the impact of price limits on price discovery, volatility, and liquidity. Some researchers used similar methods to test the influence of price limits in other markets [10, 20–22]. However, this method also has inevitable shortcomings, as there may be significant differences in the economic fundamentals behind stocks that have reached limits and those that are about to reach limits, and even if the stock has not reached the limits, its price trend may still be affected by the limits [4, 13]. Therefore, the conclusions drawn by comparing the differences between stocks that have reached the limits and those that are about to reach limits may be biased. In addition, some studies have examined the implementation effect of price limits by comparing the differences in volatility, price trends, and trading volume of stocks in the same market before and after the implementation of price limits [4, 9]. However, since the periods before and after the implementation of price limits are different, factors such as the economic fundamentals, trading systems and rules, investor structure that affect the stock market may have undergone significant changes [13], and the results may not be completely convincing. In recent years, some researchers have attempted to study the effectiveness of price limits using cross-listed stocks [13]. Cross-listed stocks are shares from the same company but listed on different stock exchanges. This method still has strong endogeneity issues, such as the overall rationality of market investors, regulatory environment, and information environment, all of which can affect stock price fluctuations.

So far, due to limitations in sample selection and research methods, there is still controversy over whether price limits actually stabilize the market.

## 4. Research hypotheses

The purpose of implementing price limits in the securities market was to control the drastic fluctuations of stock prices and achieve market stability. However, in fact, many studies have found that price limits instead reduce market efficiency. Kim and Rhee (1997) [8] were the first to summarize the three interrelated negative effects of price limits: delayed price discovery, volatility spillover, and trading interference.

If the equilibrium price is outside the price limit, the process of price discovery is forced to pause, and it needs to wait until the next trading day for the new price limit to determine before continuing to approach the equilibrium price, which is the effect of delayed price discovery [18]. Price limits prevent stock prices from promptly correcting imbalances between supply and demand on that day, and the process of price discovery needs to be completed on subsequent trading days, increasing the volatility of stock prices in the following trading days, that is, the existence of volatility spillover [18, 19]. Price limits cause prices to be unable to reach equilibrium, and trading can only be suspended, causing imbalances in supply and demand, which forces investors to delay trading until subsequent trading days, resulting in trading interference.

Kim and Rhee (1997) [8] used trading data from the Tokyo Stock Exchange to compare the differences in price trends, volatility changes, and trading volume between stocks that had reached the price limit and stocks that had a larger price increase but had not reached the price limit in the subsequent trading days to verify whether the three hypotheses about price limits were valid. Their results showed that price limits did delay the discovery of stock prices, caused volatility to spill over, and interfered with stock trading. In addition, Bildik and Gulay (2006) [22] used data from the Istanbul Stock Exchange and Henke and Voronkova (2005) [21] used data from the Warsaw Stock Exchange to conduct studies that found similar results.

For the Chinese stock market, Chen and Long (2003) [10] used the same method as Kim and Rhee (1997) [8] and found that price limits in China’s stock market also caused delayed price discovery, volatility spillover, and trading interference. However, Kim, et al. (2013) [4] used data from the period of no price limit restrictions from 1992 to 1996 and the period of implementing the price limit from 1996 to 2000 in the Chinese stock market to test the effectiveness of price limits in China stock market. They selected stocks that had a price increase of 10% when there were no price limits as the benchmark group and compared them with stocks that had reached the price limit after the price limit was implemented. Their research showed that the price limit helped with the discovery of stock prices, alleviated price volatility, and reduced abnormal trading. Overall, they believe that China’s stock market price limit system is effective. In addition, Li, et al. (2014) [12] used cross-listed stocks and found that the price limit system in China’s stock market did not cause delayed price discovery, but it did cause trading interference and volatility spillover.

Due to different data selection and research methods, there is no consensus on whether the hypotheses of delayed price discovery, volatility spillover, and trading interference related to price limit systems are valid.

In addition to the three hypotheses mentioned above, there are studies suggesting that the price limit system may have a magnet effect [7, 23–27]. The magnet effect refers to the phenomenon where the stock price accelerates towards the limit price when it approaches the daily limit, as if the limit is a magnet that attracts the stock price towards its boundary. Subrahmanyam (1994) [28] argued that the magnet effect has two underlying causes: illiquidity and behavioral investors. When the stock price approaches the limit price, behavioral investors are afraid of losing liquidity and rush to sell or buy stocks, leading to an acceleration of the stock price towards the limit.

We conduct an empirical research on the four hypotheses of price limits: the delayed price discovery hypotheses, the volatility spillover hypotheses, the trading interference hypotheses, and the magnet effect hypotheses to evaluate the effectiveness of the price limit system.

## 5. Empirical findings

### 5.1. Data and sample

Starting from August 24th, 2020, the daily price limit for regular trading of non-ST stocks on China’s ChiNext market was expanded from 10% to 20%. This was the first adjustment made to the price limit system since its implementation in 2009. We use the pre-reform events where stock closing prices hit price limits and post-reform events where stock closing prices changed beyond the 10% threshold to study the effectiveness of price limits. To avoid the impact of the policy adjustment during the transition period and ensure that the economic fundamentals did not change significantly due to long period of time, we further limit the event occurrence time to the six-month period before and after the adjustment of the price limit system, from February 1, 2020 to July 31st, 2020, and from October 1st, 2020 to March 31, 2021, respectively. Companies listed after August 24th, 2020, were all listed through the registration-based IPO system which is different from the previous listing system. To avoid the impact of different listing systems, we exclude ChiNext stocks listed after August 24th, 2020. To avoid the impact of initial listing factors, data with less than 100 trading days before the event day are also excluded. Daily trading data for stocks and the market are obtained from the China Stock Market & Accounting Research (CSMAR) database, and some data were supplemented using the Wind database.

Table 1 shows the number of events where the closing prices reached the price limits before the reform and the number of events where the closing price exceeded the 10% threshold after the reform. Before the reform, there were 1537 events of upper limit hits and 1042 events of lower limit hits. While after the reform, there were 942 events of closing price increases exceeding the 10% threshold and 329 events of closing price decreases exceeding the 10% threshold. It can be seen that, regardless of whether it was before or after the reform, the number of upper limit-hit events was far greater than that of lower limit-hit events. There is another phenomenon worth noting, which is the significant difference in the change proportion of limit-up and limit-down events after the reform. The number of limit-up events decreased from 2530 to 2406, with a decline of 38.71% (i.e., (1537–942)/1537×100%). In contrast, the number of limit-down events decreased from 1210 to 774, with a significant decline of 68.43% (i.e., (1042–329)/1042×100%). These results preliminarily suggest that the impact of relaxing the price limit system is asymmetric, and it may have a greater impact on stock price declines.

**Table 1:** Statistics of limit hit or 10% threshold hit events on the ChiNext market.

Upper limit hit or upper threshold hit		Lower limit hit or lower threshold hit	
2020.2.1–020.7.31	2020.10.1–020.3.31	2020.2.1–020.7.31	2020.10.1–2020.3.31
N = 1537	N = 942	N = 1042	N = 329

### 5.2. Delayed price discovery hypothesis

We use the event study methodology to investigate whether price limits lead to delayed price discovery. The event days before the price limits reform of China’s ChiNext market are defined as trading days where the closing price hits the limit. The event days after the price limits reform are defined as trading days where the closing price change exceeds the 10% threshold. Day 0 represents the event day. Day -1 represents the day before the event day. Day 1 represents the day after the event day, and so on for other trading days.

The daily abnormal return is estimated using the market model, and the daily market return is calculated as the weighted average daily return of all ChiNext stocks based on their market capitalization. The estimation window is from day -120 to day -30. The event window is from day -5 to day 10. Events with less than 60 trading days in the estimation window are excluded. First, the parameters αi and βi are estimated using the data from the estimation window based on Eq (1). Then, the daily abnormal return *AR*_*it*_ for stock *i* on trading day *t* in the event window is calculated based on Eq (2), and the cumulative abnormal return *CAR*_*it*_ from day -5 to day *t* is calculated based on Eq (3). Next, the mean daily abnormal return *AR*_*t*_ and the mean cumulative abnormal return *CAR*_*t*_ for each day from day -5 to day 10 before and after the price limits reform are calculated separately using Eqs (4) and (5) for both upper limits and lower limits. *AR*_*t*_ and *CAR*_*t*_ before and after the reform are then compared to determine if there is a significant difference in the price discovery process.


$$R_{it}=\alpha_{i}+\beta_{i}R_{mt}+\varepsilon_{it}$$ (1)

$$AR_{it}=R_{it}-({\hat{\alpha }}_{i}+{\hat{\beta }}_{i}R_{mt})$$ (2)

$$CAR_{it}=\sum_{i=-5}^{t}AR_{it}$$ (3)

$$AR_{t}=\frac{1}{N}\sum_{i=1}^{N}AR_{it}$$ (4)

$$CAR_{t}=\frac{1}{N}\sum_{i=1}^{N}CAR_{it}$$ (5)

If the price limit does cause a delay in price discovery, it is expected that before the reform, the daily abnormal return following the event day will continue the trend of stock returns on the event day. However, after the reform of relaxing price limits, the daily abnormal return will no longer continue the trend or the intensity of the trend will be significantly smaller than before the reform.

Table 2 shows the calculated mean daily abnormal return of stocks from day -5 to day 10 before and after the limit system adjustment in the "rise" and "fall" scenarios using the method described earlier.

**Table 2:** Mean daily abnormal returns surrounding limit-hit days or threshold-hit days.

	Up			Down		
Day	Before(N = 1537)		After(N = 942)	Before(N = 1042)		After(N = 329)
-5	0.0007		-0.0009	0.0019**		0.0045*
-4	0.0011*	>>	-0.0003	-0.0004	<<<	0.0105***
-3	0.0026***	>	0.0009	0.0013	<<<	0.0119***
-2	0.0028**		0.0031***	0.0051***		0.0041
-1	0.0039***		0.0044***	-0.0010		-0.0033^
0	0.0868***	<<<	0.1389***	-0.0357^^^	>>>	-0.1149^^^
1	0.0024***	>>>	-0.0076^^^	-0.0337^^^	<<<	-0.0068^^^
2	-0.0025^^		-0.0019	-0.0050^^^		-0.0077^^^
3	-0.0040^^^		-0.0036^^^	-0.0029^^^		-0.0037^^^
4	-0.0007		-0.0024^^	0.0058**	>>	0.0023*
5	-0.0034^^^		-0.0044^^^	0.0006		0.0006
6	0.0002	>>	-0.0026^^	-0.0005		-0.0007
7	0.0009	>>>	-0.0037^^^	-0.0024^^		-0.0011
8	-0.0007	>	-0.0029^^	0.0007	>	-0.0023
9	-0.0009	>	-0.0032^^	0.0013*	>>	-0.0025
10	-0.0001	>>	-0.0037^^^	0.0008		0.0013

Note: This table presents the mean daily abnormal returns surrounding day 0 when stocks experience price-limit hits or 10% threshold hits before and after the price limit reform of China’s ChiNext market. The daily abnormal return is estimated using the market model, and the market return is calculated as the weighted average return of all ChiNext stocks based on their market capitalization. Day 0 represents the event day. Day -1 represents the day before the event day. Day 1 represents the day after the event day, and so on for other trading days. *, **, and *** (^, ^^, and ^^^) indicate that the value is significantly greater (smaller) than 0 at the 1%, 5%, and 10% levels, respectively. >, >>, and >>> (<, <<, and <<<) indicate that the left-hand value is significantly greater (smaller) than the right-hand value at the 1%, 5%, and 10% levels, respectively, using two-sample t-test.

First, looking at the scenario where stock prices rise, it can be seen that the mean daily abnormal return before the reform on day 0 is 8.68%, which is smaller than the mean daily abnormal return after the reform at 13.89%. This is an inevitable result of the research design because the return rate on day 0 before the reform is restricted to be no more than 10%, and the stock return rate on day 0 after the reform is greater than or equal to 10%. Therefore, the daily abnormal return after the reform on the event day is expected to be greater than the daily abnormal return before the reform on the event day. The research focuses mainly on the situation after day 0. On day 1, the left value is significantly greater than the right value. Before the reform, the mean daily abnormal return on day 1 is 0.24%, which is significantly greater than 0 at 1% level. Therefore, it does continue the trend of day 0. On the other hand, after the reform, the mean daily abnormal return on Day 1 is -0.76%, which is significantly negative and opposite to the trend of the stock price on day 0, indicating that the market has an overreaction on day 0 and the stock price has since been corrected in the opposite direction. The comparison of abnormal returns on day 1 provides evidence that the stricter price limit system does indeed result in more severe delays in price discovery. However, it also mitigates to some extent the market overreaction. When comparing the values from day 2 to day 5, there is no significant difference between the pre-reform and post-reform mean abnormal return in the increase scenario. This means that the price discovery delay caused by the price limit system does not persist for a long time.

Now let’s look at the results in the scenario where stock prices fall. The difference in mean daily abnormal return before and after the reform on day 0 is the same as in the rising scenario and will not be repeated. We will also focus on the situation after the event day. It can be seen that before the reform on day 1, the mean daily abnormal return is -3.37%, significantly less than 0, and undoubtedly continues the trend of day 0. After the reform, although the stock price trend on day 1 continues to be negative, it is only -0.68%, which is far less than the -3.37% before the reform. These results indicate that in the falling scenario, stricter price limit system does indeed cause a more serious delay in price discovery, and the price limit system is inefficient. When comparing the values from day 2 to day 8, there is no significant difference between the pre-reform and post-reform abnormal return in the falling scenario. This means that the price discovery delay caused by the price limit system does not persist for a long time.

**Fig 1:**
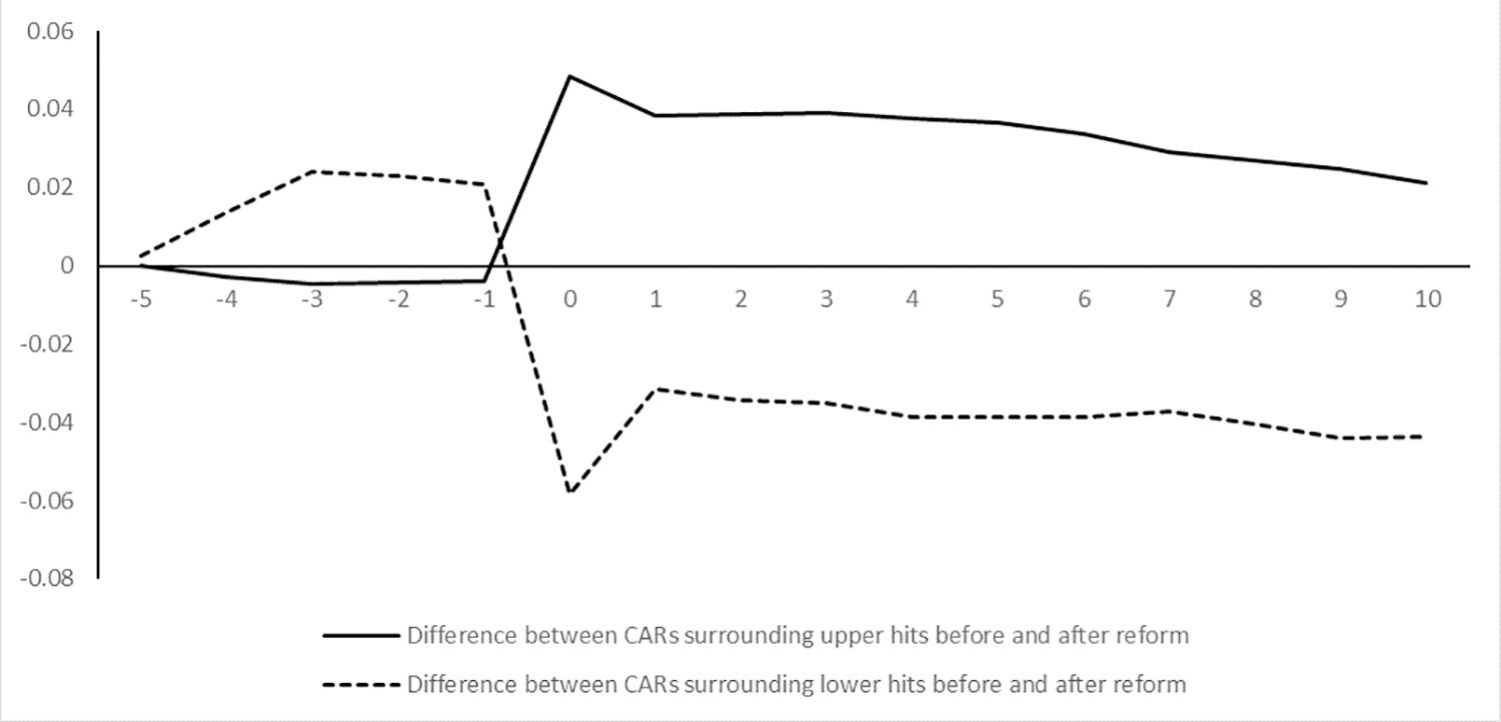
Changes in the difference of cumulative abnormal return.

To better display the trend of abnormal return before and after the reform, Fig 1 shows the difference in mean cumulative abnormal return before and after the reform from day-5 to day10 in the rising and falling scenarios. It can be seen that in the rising scenario, the difference in mean cumulative abnormal return before and after the reform reaches the maximum on day 0, followed by a significant decline on day 1. In the falling scenario, after the difference in mean cumulative abnormal return reaches its maximum value on day 0, it has a significant increase on day 1 in the opposite direction. The magnitude of the change on day 1 in the scenario of stock price rising is greater compared to that in the scenario of stock price rising.

The above analysis indicates that the price limit system indeed causes a delay in price discovery, and the delay is more severe during the process of stock price decline.

### 5.3. Volatility spillover hypothesis

The calculation of daily stock volatility, referring to Li, et al. (2014) [12], Grossman (1988) [29], is as follows:

$$Vol_{it}=\ln(H_{it}/L_{it})$$ (6)

where *H*_*it*_ is the highest stock trading price on day *t*, and *L*_*it*_ is the lowest stock trading price on day *t*. To ensure that volatility can be compared across different periods, we normalize the volatility of trading days from day -1 to day 10 by dividing it by the mean volatility of day -5 to day -2 [4, 12].

If price limits indeed cause volatility spillover, we expect the volatility after the limit day before the price limits reform to be significantly higher than that after the relaxation of price limits. Table 3 presents the mean standardized daily volatility from day -1 to day 10 before and after the reform.

**Table 3:** Daily volatility surrounding limit hit days or threshold hit days.

	Up			Down		
Day	Before(N = 1537)		After(N = 942)	Before(N = 1042)		After(N = 329)
-1	1.1758***		1.1610***	1.2049***		1.1976***
0	1.8346***	<<<	2.8431***	0.6873^^^	<<<	2.4106***
1	1.6567***	>>>	1.5259***	1.8206***	>>>	1.2718***
2	1.4163***		1.4344***	1.2539***	>>>	1.0652**
3	1.3042***		1.2847***	1.1778***	>>>	1.0041
4	1.3092***		1.2577***	1.1781***	>>>	0.9279^^^
5	1.2595***	>	1.1956***	1.1853***	>>>	0.9734
6	1.2201***		1.2069***	1.0827***	>>	0.9707
7	1.2200***		1.2225***	1.0446**	>>	0.9458^^
8	1.2075***		1.2157***	1.0551**	>	0.9752
9	1.2277***		1.1831***	1.0812^	>>	0.9299^^^
10	1.2025***	>	1.1425***	1.1598***	>>>	0.9629^

Note: This table presents the mean standardized daily volatility surrounding Day 0 when stocks experience price limit hits before the reform or 10% threshold hits after the reform. Daily volatility is measured as log of the ratio of the highest price to its lowest price. Each day’s volatility is standardized by the average volatility from day -5 to day 2. Day 0 represents the event day. Day -1 represents the day before the event day. Day 1 represents the day after the event day, and so on for other trading days. *, **, and *** (^, ^^, and ^^^) indicate that the value is significantly greater (smaller) than 0 at the 1%, 5%, and 10% levels, respectively. >, >>, and >>> (<, <<, and <<<) indicate that the left-hand value is significantly greater (smaller) than the right-hand value at the 1%, 5%, and 10% levels, respectively, using two-sample t-test.

On Day 0, the mean standardized daily volatility before the reform of the price limits is much lower than that after the reform, which is a result of the research design. Before the reform, there is a limited range of variation, restricted to within 10%. The relaxation of the price limits after the reform leads to a larger range of stock price fluctuations, causing the volatility after the reform to be greater than that before the reform on day 0.

The research primarily focuses on the trading days after day 0. It can be observed that on day 1, regardless of the increase or decrease scenario, the mean standardized daily volatility before the system adjustment is much higher than that after the reform, indicating that stricter price limits do lead to more severe volatility spillover. When comparing the values on day 2 and onwards, there is no significant difference between the pre-reform and post-reform volatility in the increase scenario. However, in the decrease scenario, the mean standardized daily volatility before the reform is much higher than that after the reform from day 2 to day 10.

These results suggest that the volatility spillover caused by price limits is asymmetric in the increase and decrease scenarios, with more severe spillover and a wider range in the case of a stock price decline. The finding supports the hypothesis of volatility spillover caused by price limits, which is opposite to the conclusion drawn by Kim, et al. (2013) [4] based on data from the Chinese stock market from 1992 to 2000.

### 5.4. Trading interference hypothesis

We standardize the daily trading volume of each stock from day -1 to day 10 by dividing the daily trading volume of each stock by the mean daily trading volume from day -5 to day -2 [4, 8, 10, 12].

According to the trading interference hypothesis, the price limit system restricts the range of price movements, preventing stock prices from reaching equilibrium prices in a timely manner. This results in a supply-demand imbalance and forces investors to delay their trades, leading to reduced trading volume on event days followed by increased trading volume in subsequent days. If the price limit system does indeed cause trading interference, it is expected that the pre-reform trading volume on day 0 will be significantly lower than the post-reform trading volume on day 0, and the pre-reform trading volume is expected to be significantly higher than the post-reform trading volume on days following the event days. Table 4 presents the mean standardized daily trading volume from day -1 to day 10 before and after the reform.

**Table 4:** Daily trading volume surrounding limit hit days or threshold hit days.

	Up			Down		
Day	Before (N = 1537)		After (N = 942)	Before (N = 1042)		After (N = 329)
-1	1.0908***		1.1192***	1.1138***		1.1056***
0	1.7987***	<<<	2.7905***	0.6930^^^	<<<	2.0596***
1	3.0383***	>>	2.7831***	1.3576***		1.3798***
2	1.9099***	<<<	2.1749***	1.1959***	>>	1.1081***
3	1.7244***	<<<	1.9066***	1.0510***	>>	1.0048
4	1.5827***	<<<	1.7718***	1.1417***	>>>	0.9364^^
5	1.4941***	<<<	1.6446***	1.1132***	>>>	0.9146^^^
6	1.4565***	<<	1.5757***	1.0138	>	0.9379^^
7	1.4635***	<<	1.5675***	1.0282	>>>	0.8976^^^
8	1.4827***		1.5525***	1.0821***	>>>	0.9174^^
9	1.4365***		1.4952***	0.9911	>>	0.8832^^^
10	1.4181***		1.4403***	1.1773***	>>>	0.9107^^^

Note: This table presents the mean standardized daily trading volume surrounding day 0 when stocks experience price-limit hits before the reform or 10% threshold hits after the reform. Daily trading volume is measured as number of shares traded. Each day’s trading volume is standardized by the average daily trading volume from days -5 to -2. Day -1 represents the day before the event day, and Day 1 represents the day after the event day, and so on for other trading days. *, **, and *** (^, ^^, and ^^^) indicate that the value is significantly greater (smaller) than 0 at the 1%, 5%, and 10% levels, respectively. >, >>, and >>> (<, <<, and <<<) indicate that the left-hand value is significantly greater (smaller) than the right-hand value at the 1%, 5%, and 10% levels, respectively, using two-sample t-test.

Let’s first look at the results of upper limit hits and upper threshold hits. As we can see, on day 0, regardless of before or after the reform, the mean standardized daily trading volume is significantly higher than the previous day (t-test also shows that the mean standardized daily trading volume on day 0 before and after the reform is significantly higher than that on day -1 at the 1% level). Moreover, on day 0, the mean standardized daily trading volume is significantly lower than that after the reform. On day 1, the mean standardized daily trading volume before the reform increases to 3.0382, while the mean standardized daily trading volume after the reform decreases to 2.7381. The mean standardized daily trading volume before the reform is significantly higher than that after the reform at the 5% level, indicating that stricter price limits do cause some trading interference, leading to some delayed trades on day 1. However, comparing the trading volume from day 2 to day 7, we can see that although the trading volume before and after the reform do not return to normal levels and both are significantly higher than 1, the mean standardized daily trading volume before the reform is always significantly lower than that after the reform, indicating that the trading volume before the reform returns to the normal level faster after the event day. These results show that stricter price limits do cause more serious trading interference under the situation of rising stock prices, but the interference is not significant, only causing an increase in trading volume for one trading day, and making the subsequent trading volume recover to normal levels faster, reflecting some effectiveness.

As for the situation of stock price decline, there is no significant difference in trading volume on day 1. However, compared to the value on day 0, the mean standardized daily trading volume before the reform increases from 0.6930 to 1.3576, while the mean standardized daily trading volume after the reform decreases from 2.0596 to 1.3798, indicating that the trading before the reform is more seriously interfered with on the event day. Starting from day 2, the mean standardized daily trading volume before the reform is significantly higher than that after the reform. Additionally, the mean standardized daily trading volume before the reform is always significantly higher than 1 from day 1 to day 5, while the mean standardized daily trading volume after the reform falls below 1 from day 3, indicating that the trading volume before the reform recovers to normal levels slower. The above analysis shows that stricter price limits do cause more serious trading interference in the situation of falling stock prices.

From the above analysis, we can see that price limits do indeed have some interference with trading, but the strength of their effect may differ in the situations of stock price increases and decreases. In the situation of a stock price decline, the interference effect of price limits on trading is stronger.

### 5.5. Magnet effect hypothesis

We examine the distribution of large price movements before and after the reform to determine whether price limits have a magnet effect [4]. Specifically, we identify the number of occurrences where a stock’s daily highest (lowest) price has an increase (decrease) relative to the previous day’s closing price in the following ranges: 5%-5.99%, 6%-6.99%, 7%-7.99%, 8%-8.99%, 9%-9.99%, and 10% or more. Then we calculate the percentage of each large price movement category, which is equal to the number of days with the stated large price movement divided by the total number of days of all large price movements. We compare the differences in these proportions before and after the reform.

According to the hypothesis of the magnet effect, when the stock price approaches the limit price, it will accelerate towards the limit price and eventually reach it, just like a magnet attracting metal. If the magnet effect exists, then the proportion of events where the price movement is slightly below the limit will be very small. In the current research, this means that the proportion of events where the price movement is between 9%-9.99% before the reform will be significantly smaller than that after the reform. Table 5 presents the statistical results.

**Table 5:** Comparison of the proportion of large price changes.

Price Movement	Up			Down		
	Before		After	Before		After
≥10%	0.1372	<<<	0.2033	0.0958	>>	0.0869
9%-9.99%	0.1996	>>>	0.0696	0.1742	>>>	0.0470
8%-8.99%	0.0957		0.0985	0.0975	>>>	0.0819
7%-7.99%	0.1274	<<	0.1355	0.1428		0.1445
6%-6.99%	0.1752	<<<	0.2020	0.2037	<<<	0.2340
5%-5.99%	0.2649	<<<	0.2911	0.2860	<<<	0.4057

Note: This table presents the percentage of days when the price change from the previous day’s closing price to the next day’s highest or lowest price falls within the ranges of 5%–5.99%, 6%–6.99%, 7%–7.99%, 8%–8.99%, 9%–9.99%, and 10% or more. The reported percentage equals the number of days with the stated price movements divided by the total number of days with all large price movements. <, <<, and <<< (>, >>, and >>>) indicate that the left-hand value is significantly lower than the right-hand value at the 1%, 5%, and 10% levels, respectively, using two-sample Z-test of proportions.

First, in situations where the stock price increases, the proportion of events in the second interval of 9%-9.99% is 19.96% before the reform, but the proportion decreases significantly to 6.96% after the reform of the system. The proportion of events in this interval does not increase due to the relaxation of the price limit system, but instead decreases significantly. The statistical results for downward movements are the same as those for upward movements. This indicates that the magnet effect does not exist in either upward or downward movements, which is consistent with the findings of Kim, et al. (2013) [4].

## 6. Conclusions

Since the implementation of China’s 10% price limit regulation in the stock market in 1996, there has been an ongoing debate regarding its effectiveness in market stabilization. In August 2020, the price limits in the ChiNext market were adjusted for the first time, increasing from 10% to 20%. Utilizing this reform, this research tests the effectiveness of China’s price limit system.

The research concentrates on four hypotheses related to the adverse effects of price limits, namely the price discovery delay hypotheses, volatility spillover hypotheses, trading interference hypotheses, and magnet effect hypotheses. Contrary to previous findings [4], the empirical results indicate that price limits in China’s stock market do cause price discovery delay, volatility spillover and trading interference. Additionally, we find that these effects are asymmetric in the upward and downward price movements, with more serious negative effects under the downward movement. However, the research finds no evidence of the magnet effect of price limits.

These findings have important reference value for further reform of the price limit system in China’s stock market. The subsequent arrangement of the system can consider an asymmetric structure, which allows for a wider range of price declines compared to price increases.
